# The Dynamics of Signal Triggering in a gp130-Receptor Complex

**DOI:** 10.1016/j.str.2007.02.006

**Published:** 2007-04

**Authors:** Rishi Matadeen, Wai-Ching Hon, John K. Heath, E. Yvonne Jones, Stephen Fuller

**Affiliations:** 1 Division of Structural Biology, The Wellcome Trust Centre for Human Genetics, University of Oxford, Roosevelt Drive, Headington, Oxford OX3 7BN, United Kingdom; 2 Cancer Research UK, School of Biosciences, University of Birmingham, Edgbaston, Birmingham B15 2TT, United Kingdom

**Keywords:** MOLIMMUNO, SIGNALING

## Abstract

gp130 is a shared signal-transducing membrane-associated receptor for several hematopoietic cytokines. The 30 Å resolution cryo-electron microscopy (cryo-EM) structure of the Interleukin 11(IL-11)-IL-11 Receptor-gp130 extracellular complex reveals the architecture and dynamics of this gp130-containing signaling complex. Normal-mode analysis reveals a repertoire of conformational changes that could function in signal triggering. This suggests a concerted mechanism of signaling involving all the components of the complex. This could provide a general mechanism of signal transfer for cytokines utilizing the JAK-STAT signaling cascade.

## Introduction

The cell-surface receptor gp130 transduces signals involved in the regulation of a wide variety of adult tissue systems, including the hematopoietic system, nervous system, bone, heart, adipose tissue, testes, liver, and muscle (Bravo and Heath, 2000). Signaling results from the extracellular, ligand-mediated formation of oligomeric receptor complexes. These complexes can differ in composition and stoichiometry depending upon the ligand, but they require the incorporation of one or more molecules of gp130 (Hibi et al., 1990). The ligands are termed the gp130-binding cytokines and include Interleukin 6 (IL-6), Interleukin 11 (IL-11), Ciliary Neurotrophic Factor (CNTF), Oncostatin M (OSM), and Leukemia-Inhibitory Factor (LIF) (Kishimoto et al., 1995; Taga and Kishimoto, 1997). IL-6 and IL-11 signaling occurs via homodimerization of gp130 after the formation of a hexameric complex containing two molecules of gp130, two molecules of IL-6 or IL-11 ligand, and two molecules of a specific, nonsignaling IL-6 or IL-11 receptor (termed IL-6R and IL-11R, respectively) (Ward et al., 1994; Paonessa et al., 1995; Barton et al., 2000).

Crystal structures of the gp130-binding cytokines IL-6, LIF, CNTF, and OSM have revealed a highly conserved four-α helix bundle topology (Bravo and Heath, 2000). The sequences of gp130, and other cognate receptors for the four-α helix bundle cytokines, are distinguished by a tandem pair of distinctive fibronectin type III (FNIII)-like domains in their N-terminal extracellular regions (Cosman, 1993). This is termed the cytokine-binding homology region (CHR). Crystal structures have been determined for the CHR of gp130 (Bravo et al., 1998), the entire extracellular region of IL-6R (Ig-like plus cytokine-binding domains) (Varghese et al., 2002), and for complexes involving soluble fragments of gp130 (Chow et al., 2001; Boulanger et al., 2003a, 2003b). These structural analyses, combined with functional studies, have provided significant insights into the key features and characteristics of the interaction surfaces required for affinity and specificity in gp130-binding cytokine-receptor recognition.

Many of the remaining questions concerning the triggering of signaling by gp130-binding cytokines now relate to the larger-scale architecture and dynamics of the cytokine-receptor assemblies. The contribution of the N-terminal Ig-like domain of gp130 to the formation of the IL-6 signaling complex has been addressed by crystal structures from Garcia and coworkers (Chow et al., 2001; Boulanger et al., 2003b). However, little structural data are available to illuminate the role of the three FNIII-like domains that are predicted by sequence analysis to form the C-terminal half of the gp130 extracellular region. Biophysical data have indicated that interactions involving these domains do contribute to complex formation (Boulanger et al., 2003b). Furthermore, functional data point to a clear role for the FNIII-like domains of gp130 in signaling (Timmermann et al., 2002).

The complete extracellular portion of a gp130-binding cytokine-receptor signaling complex has evaded crystallization. Lower-resolution EM studies provide an alternative route for structural analysis of the gp130-binding cytokine system (Skiniotis et al., 2005). Cryo-electron microscopy (cryo-EM) in particular allows for the investigation of the structural dynamics. We have used this approach for the entire extracellular region of the hexameric signaling complex formed by IL-11. IL-11 signals through a complex comprising a 2:2:2 stoichiometry of IL-11, IL-11R, and gp130 (Barton et al., 2000). In the following sections, we refer to the soluble form of this complex as IL-11H. Our single-particle cryo-EM reconstruction of IL-11H provides the first, to our knowledge, complete native structure for the extracellular portion of a gp130-binding cytokine signaling complex in vitrified water. The reconstruction reveals a distinct head and two leg regions. The cryo-EM map is used to construct a discrete map via the techniques of vector quantization. This model structure, derived directly from the cryo-EM reconstruction, has enabled the calculation of normal modes of the complex, which allows us to catalog the conformational changes that may participate in cellular communication. In addition, the architecture of the cryo-EM map convincingly accommodates a modeled crystal structure of the hexameric IL-6 signaling complex (which includes the membrane-distal half of gp130 [Boulanger et al., 2003b]), plus two “beads on a string” arrangements of FNIII domains (consistent with the membrane-proximal halves of two copies of gp130). The combination of the cryo-EM and normal-mode analyses allows the correlation of the density distribution in the cryo-EM map to the calculated dynamical properties. This provides a novel insight into the mechanism of signal triggering, relevant to several gp130-containing receptor complexes.

## Results

### Cryo-EM Structure of IL-11H

**Figure 1:**
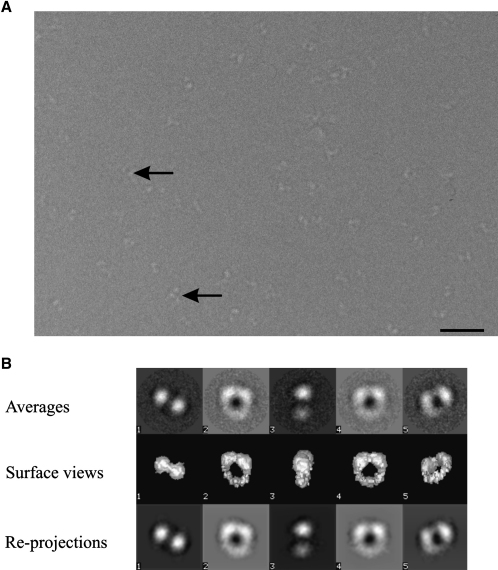
Cryo-EM of the IL-11-IL-11R-gp130 Complex (A) Part of a typical electron micrograph of the purified IL-11-IL-11R-gp130 complex. Arrows identify examples of the complexes within vitreous ice. The scale bar represents 50 nm on the specimen scale. (B) A summary of the three-dimensional image analysis. Characteristic class averages obtained by multireference alignment and classification are shown in row 1. Surface views from the three-dimensional structure of the IL-11-IL-11R-gp130 complex, in the Euler directions assigned to the averages, are shown in row 2. Row 3 contains reprojected images from the three-dimensional structure in the Euler directions found for the corresponding averages.

The soluble IL-11H complexes were prepared as described in Experimental Procedures. A summary of the image analysis is shown in Figure 1. A total of 830 images were extracted from 15 micrographs and were subjected to single-particle analysis as described in Experimental Procedures.

**Figure 2:**
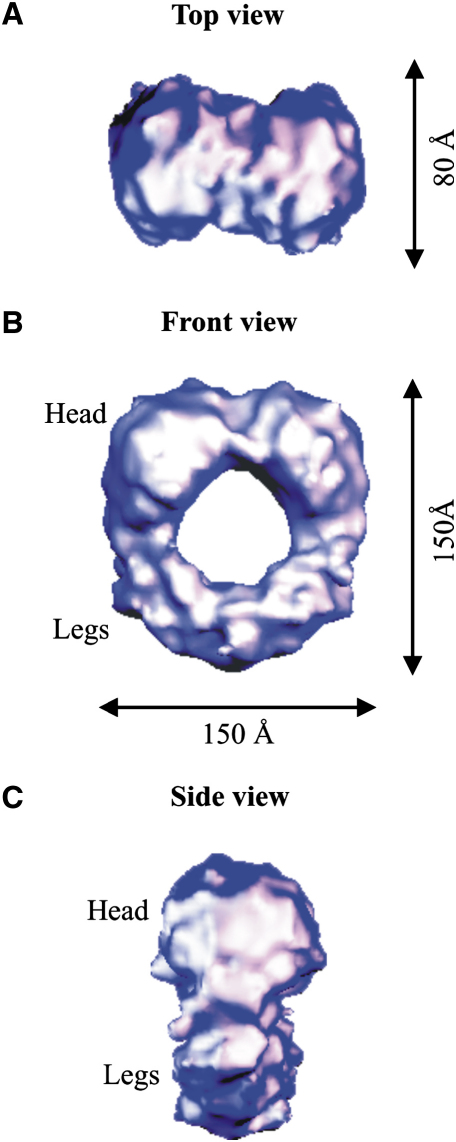
Orthogonal Views of the IL-11H Complex (A–C) The (A) top, (B) front, and (C) side views of the IL-11H complex are contoured at 2.5σ.

**Figure 3:**
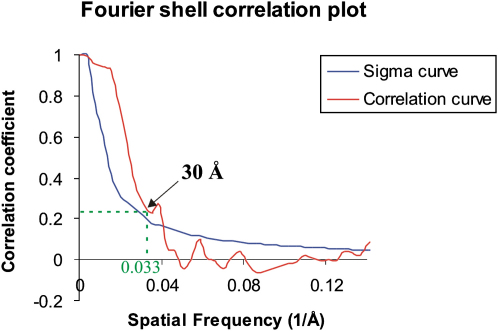
Fourier Shell Correlation Plot from the IL-11H Data Set Fourier shell correlation curve from the IL-11H data set is shown in red plot. The 3σ threshold curve is multiplied by √2 = 1.41 to account for the two-fold redundancy of the C2 pointgroup symmetry of the data (blue curve). The resolution of the reconstruction is ∼30 Å (corresponding to a spatial frequency of 0.033 Å^−1^). The position of 30 Å resolution is marked.

The extracellular portion of IL-11H is a flat, ring-shaped complex (Figures 2A–2C). The dimensions of the hexamer are 150 Å × 150 Å × 80 Å, and the central hole is 50 Å in diameter (Figures 2A and 2B). The top of the complex (head, Figure 2) is larger than the bottom (legs, Figure 2B). The two-fold symmetric (C2) structure comprises identical subunits that twist toward a point of contact at the bottom of the molecule (Figures 2B and 2C). The two-fold axis of symmetry runs from the head across the central hole to the bottom of the legs. The resolution of the reconstruction is 30 Å, as estimated from the spatial frequency intersection of the Fourier shell correlation (FSC) plot and the 3σ threshold function corrected for the C2 point group symmetry (Orlova et al., 1997) (Figure 3). A resolution of 30 Å is also predicted by using a 0.2 crosscorrelation threshold (Rosenthal and Henderson, 2003). The drop in the FSC plot is steep, indicating that the resolution estimate is robust and that the number of images used in the analysis is sufficient. The correlation coefficient of the projections of the map and its enantiomer is 0.97, indicating a weakly handed structure (van Heel et al., 1996).

### Flexibility in the Cryo-EM Map

**Figure 4:**
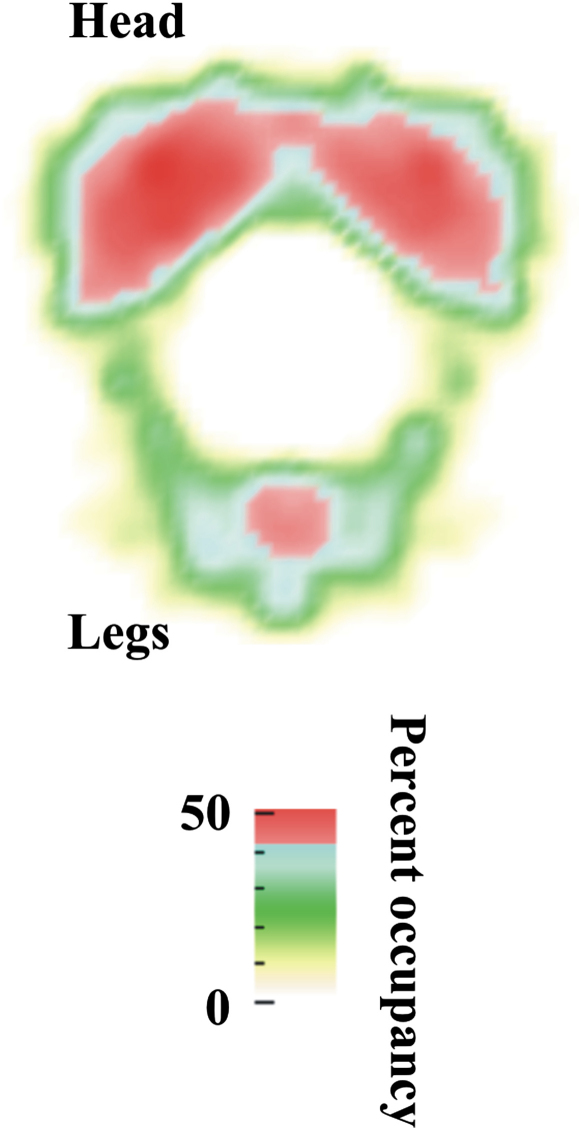
Density Distribution in the Cryo-EM Map Percent occupancy is defined as the density distribution relative to the highest density in the map. The density is strongest at the head and central region of the legs. The region connecting the head and legs shows lower density, suggesting greater flexibility.

The electron potential of the structure gives rise to the observed density in a cryo-EM reconstruction. The strength of the observed density is modulated by variation in flexibility between the particles incorporated into the reconstruction. In the IL-11H cryo-EM structure, the head of the molecule is of stronger density than the legs (Figure 4). However, a relatively strong density is seen where the two protruding legs meet. Weak density areas exist between the head and legs of the complex and indicate mobile regions within the hexamer. Another weak area of density is located at the center of the head region (Figure 4).

### Elastic Normal Modes of the Discretized Cryo-EM Map

The cryo-EM map was vector quantized to create a discrete, reduced description of its continuous shape/mass distribution (Wriggers et al., 1999). The docking program Situs (Wriggers et al., 1999) was used for the vector quantization of the cryo-EM electron density. A total of 50 so-called codebook vectors were chosen to describe the cryo-EM map, a sufficient number for reconstructions at a relatively low resolution (∼30 Å) (Tama et al., 2002) (see Experimental Procedures). This discrete elastomechanical model for IL-11H was then subjected to normal-mode analysis (NMA). This technique has been shown to be very useful in the study of protein motions (Tama and Sanejouand, 2001). NMA provides a repertoire of possible conformational changes based on the discrete model calculated from the cryo-EM map. We can use this information as a complement to cryo-EM to visualize the dynamics of the structure. The extent of functionally important large-scale rearrangements of molecular structures and assemblies is well represented by the lowest-frequency normal modes (Tama et al., 2003). *ElNémo*, the web interface to the Elastic Network Model, provides a tool to compute, visualize, and analyze low-frequency normal modes of large macromolecules (Suhre and Sanejouand, 2004). Several motions show potential functional significance and are supported by the flexible regions in the cryo-EM map (Figure 6; Movies S1–S3, see the Supplemental Data available with this article online). The lowest mode (mode 1, after the six global translation and rotation modes) describes the motion of the gp130 legs and reveals a change from an extended to a compressed conformation (Movie S1). These motions are also seen in modes 2 and 3 (Movies S2 and S3, respectively), although these modes are at different normalized frequencies. Modes 1 and 3 comprise a rotation around the C2 symmetry axis of the hexamer, indicating a conformational twist that could be important in positioning intracellular components. Modes 2 and 3 show a “flapping” motion of the head region. Interestingly, these dynamical properties calculated from NMA are also supported by the motions suggested by the density distribution profile of the cryo-EM map (Figure 4).

### Fitting of the IL-6 Hexameric Complex Crystal Structure into the Cryo-EM Map

There is no atomic structure of complete IL-11H; however, atomic structures are available from X-ray crystallographic analysis of several components of the hexamer and structurally related molecules. We have taken the IL-6 cytokine-binding complex (Boulanger et al., 2003b) (PDB code 1P9M) to be structurally homologous to an IL-11 cytokine-binding complex. An IL-11 model has shown a “four-helix bundle” protein fold similar to that of IL-6 (Barton et al., 1999). In addition, the extracellular domains of IL-11R and IL-6R have a 24% sequence identity (Curtis et al. 1997). FNIII repeats from two segment of human fibronectin (PDB code 1FNH) were taken to be homologous to the extracellular membrane-proximal domains of gp130 (Sharma et al., 1999). Each segment consists of three FNIII domains. These components can be fitted into the IL-11H cryo-EM map. We have initially docked the crystal structure of the IL-6 cytokine-binding complex into the head region of the IL-11H cryo-EM reconstruction by using the graphics program “O” (Kleywegt and Jones, 1997) to occupy as much of the head volume as possible. FNIII repeats from two segments of human fibronectin (Sharma et al., 1999) (PDB code 1FNH) were then inserted into the leg region of the cryo-EM map (one segment for each leg region) to represent the membrane-proximal domain of gp130. Each FNIII domain was rotated to satisfy the kink of the leg region. A model of complete extracellular IL-11H was thereby created. The fit of this hand-docked model and the cryo-EM map correlate with a coefficient of 0.54. The fit was refined by using the program URO (Navaza et al., 2002) (see Experimental Procedures). The result of the refined fit is shown in Table 1. The correlation between the observed and calculated structure factors is 0.656 and indicates a good fit; however, the R factor is high at 0.658, consistent with significant structural flexibility in the cryo-EM map compared to the modeled hexamer. Table 2 shows values calculated from contributions of the cryo-EM reconstruction exclusively around the model. Consequently, only the solid, nonflexible portions of the reconstruction are considered in the optimization procedures. The correlation between the observed and calculated structure factors is 0.918, and the R factor 0.297, indicating a good fit between the model and the nonflexible portions of the cryo-EM map.

### Composite Homology Model of IL-11H

**Figure 5:**
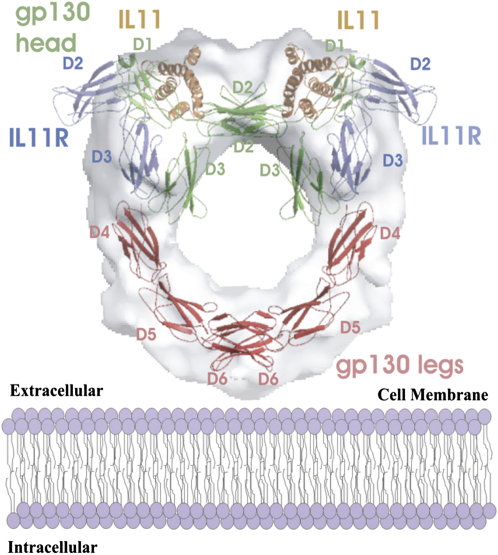
Fitting of the IL-6-IL-6R-gp130 Model into the Cryo-EM Density The cryo-EM map is shown as a gray, semitransparent surface. The fitted model is shown as a ribbon representation. The IL-6 cytokines (representing IL-11) are shown in gold, the IL-6R (representing IL-11R) D2 and D3 domains are shown in blue, the gp130 homodimer membrane-distal D1–D3 domains are shown in green (gp130 head), and the gp130 homodimer membrane-proximal D4–D6 domains are shown in red (gp130 legs). The D1 domains of IL-6R (IL-11R) are not included. The position of the cell membrane is shown by the schematic.

The modeled IL-11H and the cryo-EM map (Figure 5) have a correlation coefficient of 0.65 (Table 1). Our fit (Figure 5) places the FNIII domains representing the gp130 membrane-proximal domains in the density of the complex's legs. The legs of the gp130 homodimer meet at their respective D6 domains (Figure 4). No contact is seen between the two D5 domains, in contradiction to other models (Moritz et al., 2001). The homology models of the IL-11, IL-11R, and membrane-distal portions of gp130 fill the head region of the complex. The absence of density corresponding to D1 of the IL-11Rs could be explained by the flexibility in the linker regions between the D1 and D2 domains. The density between the two cytokine ligands is relatively weak (Figure 4). This may arise from movements in D2 of gp130.

## Discussion

The IL-6-type hematopoeitic cytokines signal via the gp130 receptor. IL-11 and IL-6 initially bind to their specific receptors, and the complex associates with membrane-bound gp130, forming the functional signaling complex. The cytokine-binding event occurs at D2 and D3 of the gp130s and is transmitted into the cell through D4, D5, and D6. The IL-11H cryo-EM map enables several regions of the functional complex to be localized by fitting a homology model. The ligand-binding region of the complex is situated at the head of the cryo-EM reconstruction (Figure 4). This region is composed of the bound cytokines, the D1–D3 domains of the specific receptors, and the D1–D3 domains of the gp130s. The signal-triggering region is located in the legs of IL-11H. This contains the membrane-proximal domains of the gp130s (D4–D6). The gp130 legs are readily located in the cryo-EM reconstruction. The legs protrude from the head to meet and cross at their respective D6 domains. This indicates that signaling is transferred to the opposite side of the molecule from where the binding event takes place. Each D6 domain leads into a transmembrane region. The close coupling of the two D6 domains in the IL-11H structure implies a close association of these gp130 transmembrane regions. The D5 domains may be important in orientating the D6 domains since deletion abrogates signal transduction (Kurth et al., 2000).

**Figure 6:**
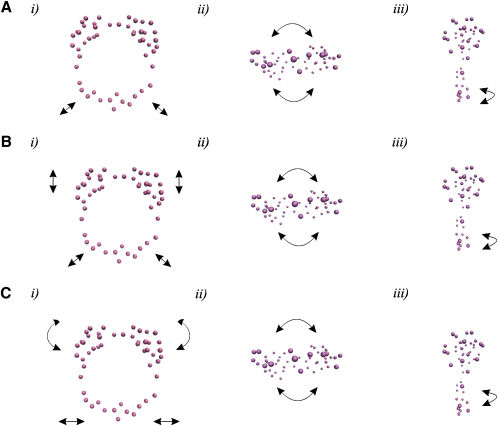
Schematic of the Movements of IL-11H Calculated from NMA Three orthogonal views (*i*, *ii*, *iii*) of the discrete map of IL-11H, composed of 50 codebook vectors (purple spheres), are shown for the three lowest-frequency normal modes (A–C). The black arrows indicate the directions of motion.

The cryo-EM of particles within vitreous ice allows for the direct imaging of the sample density. The density distributions within the cryo-EM maps obtained subsequently have information pertaining to the dynamics of the object. The cryo-EM reconstruction, together with NMA on the discrete map, has allowed us to directly calculate potential movements between these domains and correlate the pattern of motions with density distributions of the map. The discrete map is derived from vector quantization of the cryo-EM map and thus contains information pertaining directly to the IL-11R cryo-EM reconstruction. The gp130 legs have a “c-like” configuration and consequently appear compressed in the hexameric complex (Figure 6). This allows structural adaptability of the membrane-proximal domains in the height of the ligand-binding domains with respect to the cell surface, as demonstrated by NMA (Figure 6; Movies S1–S3). Indeed, this adaptability suggests that unliganded gp130 is elongated so that the positioning of the cytokine-binding domains is more distal to the membrane than it is in bound gp130. The flexibility in the gp130 legs may be an important factor in orientating the cytoplasmic intracellular domains of gp130 and their associated Janus Kinases (JAKs), promoting signal transduction (Socolovsky et al., 1999). The rotation around the C2 symmetry axis shown by NMA would facilitate this association.

A three-dimensional EM map of the related IL-6-IL-6Rα-gp130 hexameric complex in negative stain has been calculated (Skiniotis et al., 2005). This shows a related structure consisting of an elongated bipartite head domain and two leg domains, one of which is represented by a straight density and the other of which is represented by a kinked density—an arrangement that distorts the structural C2 symmetry. The distortion of the map could be due to preparation artifacts of the negative staining technique, which additionally eliminates the dynamics of the structure.

Structural and functional data for the cytokine EPO and its receptor (EPOR) have indicated that there are stringent requirements for the relative positioning of the two signaling receptors when crosslinked by the cytokine ligand if signal is to be transduced (Livnah et al., 1996, 1998). In addition the orientations of the extracellular EPOR D1 and D2 domains in the signal-competent complex provide a sensitive adjustment for the positioning of their cytoplasmic components involved in signal transduction, resulting in the modulation of the signal intensity (Wilson and Jolliffe, 1999). The signaling assemblies involving gp130 are based on a more complex architecture. Consequently, the orientations of the IL-11H extracellular domain could provide more extended, exquisite adjustments of cytoplasmic components that modulate signal intensity. These orientations are suggested by NMA through the motions and multiple configurations of the IL-11H discrete map, in particular with the movements of the gp130 membrane-proximal domains. Indeed, all of the component molecules contribute to the dynamics of the hexamer; thus, a concerted mechanism may be involved in reorientating the intracellular components into a configuration that allows for signal transduction. Initiation of the JAK/STAT pathway in response to IL-11 involves JAK-1 and predominantly STAT3 (Dahmen et al., 1998).

Cryo-EM, together with NMA, has now illuminated the relative positioning and dynamics of the three membrane-proximal FNIII-like domains in IL-11H. Furthermore, functional data point to a clear role for the FNIII-like domains of gp130 in signaling (Timmermann et al., 2002). Cytokines such as LIF, OSM, and CNTF utilize a heterodimer of gp130 and the LIF receptor (LIFR) to transduce a signal (OSM can use the OSM receptor [OSMR] instead). LIFR is similar to gp130, although it possesses an extra N-terminal (membrane-distal) copy of a cytokine-binding domain (He et al., 2005). The signaling complexes formed with these factors may have a similar conformation and dynamics in the membrane-proximal regions as the gp130s in IL-11H. Consequently, signal triggering and signal modulation could occur in a similar fashion. However, further structural and dynamic studies on these liganded receptor complexes and their unliganded receptor complexes are needed to confirm the mechanism of signal activation.

## Experimental Procedures

### Expression and Purification of the IL-11-IL-11R-gp130 Complex

Murine IL-11 (residues 22–199) was subcloned into the pET15b vector (Novagen), and the His_6_-tagged fusion protein, expressed in *E*. *coli*, was purified by metal-chelating affinity chromatography (TALON, Clonetech). The extracellular regions of murine IL-11R (residues 1–361) and gp130 (residues 1–617) were expressed as secreted, soluble, C-terminally tagged Fc fusion constructs in stably transfected *Drosophila* S2 cells and transiently transfected human epithelial kidney 293T cells, respectively. Both soluble receptors were purified from the media by adsorption to protein A Sepharose (Amersham Biosciences) and were eluted from the matrix after on-column cleavage with the rhinovirus 3C protease. The three affinity-purified subunits were mixed with IL-11 in molar excess and concentrated, and the complex was purified by gel filtration with the Superdex 200 (10/30) column (Amersham Biosciences). The purified complex used for cryo-EM imaging was in a buffer of 5 mM HEPES (pH 7.5), 100 mM NaCl, 1 mM DTT, and 0.25% β-octylglucopyranoside.

### Cryo-Microscopy and Three-Dimensional Image Processing

Purified IL-11H, at a concentration of 1 mg/ml, was applied to a holey carbon film and freeze plunged into liquid ethane after blotting off excess fluid. The vitrified samples were imaged in a Philips CM200 FEG at liquid nitrogen temperatures by using a Gatan 626 cryoholder and cryotransfer system. Images were taken at defocus values ranging from 3 to 5 μm and at an electron optical magnification of 50,000. Micrographs were digitized by using a UMAX Powerlook 3000 at a step size of 8.3 μm, corresponding to 1.67 Å on the specimen scale. This resulted in 160 × 160 pixel IL-11H images. These images were binned by a factor of 2 for final analysis, resulting in a pixel sampling distance of 3.3 Å. Image processing was performed with the IMAGIC-5 software package (van Heel et al., 1996). The full data set consisted of 830 images from 15 micrographs. The IL-11H particles were interactively selected by using the DISPLAY module. CTF compensation was performed on images as described (Matadeen et al., 1999). The reconstruction was calculated de novo; the images were first aligned by using the reference-free alignment-by-classification procedure, followed by multistatistical analysis (MSA) (van Heel, 1989). Averages of the data set were used to center the images within the data set; three rounds of iteration were performed. MSA was then used to generate class averages. Initially, 40 class averages were generated and used for further center refinement by using multireference alignment (MRA). The images were subjected to five cycles of MRA and MSA. Relative orientations of ten averaged images (those with the lowest variance) were determined by using angular reconstitution (van Heel, 1987). The orientations obtained were used to calculate a three-dimensional map by using the exact filter back-projection algorithm (Harauz and van Heel, 1986). Projections from the map were compared to their corresponding averages as a measure of the consistency of the analysis. A total of 70 projections were calculated from the map and were used in a new round of MRA followed by MSA. From 100 averages, 50 (consisting of a total of 622 particles) were selected for angular reconstitution, and a new three-dimensional map was calculated by using the resultant orientations. The process of analysis was iteratively refined until stability in alignment, resulting orientations, and FSC were observed.

### Fitting of the IL-11H Model

The X-ray crystallographic model of the IL-6 cytokine-binding complex (Boulanger et al., 2003b) together with six FNIII repeats (Sharma et al., 1999) were manually docked into the IL-11H cryo-EM map by using the program “O” (Kleywegt and Jones, 1997). The fitting refinement procedure was performed in URO (Navaza et al., 2002). This program uses an adapted rigid-body refinement to perform the fitting of molecular models to EM reconstructions in reciprocal space. Six positional variables are used in the refinement (Euler angles α, β, and γ and translational parameters x, y, and z). Data from 260 Å to 30 Å were used in the refinement procedure. Four optimization cycles were performed until no shifts in the coordinates were observed.

### Representation of the IL-11H Cryo-EM Map and Model

Volume rendering and density representation were performed in Opendx (http://www.opendx.org) and pymol (DeLano, 2002). For final representation of the results, the high-frequency components of the three-dimensional map are suppressed by a Gaussian low-pass filter with a 1/e width corresponding to ∼30 Å. Figure 4 was prepared with RASMOL (Sayle and Milner-Whilte, 1995), BOBSCRIPT (Esnouf, 1997), and RASTER3D (Merritt and Bacon, 1997). The codebook vectors were calculated by using the qvol program in Situs (Wriggers et al., 1999). A total of 50 codebook vectors were calculated, and the resultant discretized map was subjected to NMA with the *ElNemo* web server (http://igs-server.cnrs-mrs.fr/elnemo/) by using a cut-off value of 30 Å.

## Figures and Tables

**Table 1:** Result from the URO Fitting Procedure

Fitting: URO								
α	β	γ	X	Y	Z	C*f*	R*f*	F*m*
75.2	91.6	90.2	−0.0101	−0.0079	0.0178	65.6	65.8	29.6

α, β, and γ represent the Euler angles of the model; X, Y, and Z are the fractionary translations of the model; C*f* is the correlation between the observed and calculated structure factors (× 100); R*f* is the crystallographic R factor (× 100); and F*m* is the value of the optimized function (quadratic misfit) (Navaza et al., 2002).

**Table 2:** Result from the URO Fitting Procedure Calculated from Contributions of the Cryo-EM Reconstruction Exclusively around the IL-11H Model

Fitting: URO								
α	β	γ	X	Y	Z	C*f*	R*f*	F*m*
74.7	91.7	90.3	−0.0079	−0.0193	0.0175	91.8	29.7	8.0

α, β, and γ represent the Euler angles of the model; X, Y, and Z are the fractionary translations of the model; C*f* is the correlation between the observed and calculated structure factors (× 100); R*f* is the crystallographic R factor (× 100); and F*m* is the value of the optimized function (quadratic misfit) (Navaza et al., 2002).

## Accession Numbers

The IL-11H cryo-EM map has been deposited in the EM Data Bank, European Bioinformatics Institute, Cambridge with accession code EMD-1223.

## Supplemental Data

Movie S1. Dynamics of the IL11H calculated from NMA: Mode 1 Three orthogonal views of the discrete map of the IL11H, composed of 50 codebook vectors (purple spheres) are shown for each of the three lowest frequency normal modes (Movies S1, S2, and S3, respectively).

Movie S2. Dynamics of the IL11H calculated from NMA: Mode 2

Movie S3. Dynamics of the IL11H calculated from NMA: Mode 3

## References

[bib1] Barton V.A., Hudson K.R., Heath J.K. (1999). Identification of three distinct receptor binding sites of murine interleukin-11. J. Biol. Chem..

[bib2] Barton V.A., Hall M.A., Hudson K.R., Heath J.K. (2000). Interleukin-11 signals through the formation of a hexameric receptor complex. J. Biol. Chem..

[bib3] Boulanger M.J., Bankovich A.J., Kortemme T., Baker D., Garcia K.C. (2003). Convergent mechanisms for recognition of divergent cytokines by the shared signalling receptor gp130. Mol. Cell.

[bib4] Boulanger M.J., Chow D.C., Brevnova E.E., Garcia K.C. (2003). Hexameric structure and assembly of the interleukin-6/IL-6 α-receptor/gp130 complex. Science.

[bib5] Bravo J., Heath J.K. (2000). Receptor recognition by gp130 cytokines. EMBO J..

[bib6] Bravo J., Staunton D., Heath J.K., Jones E.Y. (1998). Crystal structure of a cytokine-binding region of gp130. EMBO J..

[bib7] Chow D., He X., Snow A.L., Rose-John S., Garcia K.C. (2001). Structure of an extracellular gp130 cytokine receptor signalling complex. Science.

[bib8] Cosman D. (1993). The hematopoietin receptor superfamily. Cytokine.

[bib9] Curtis D.J., Hilton D.J., Roberts B., Murray L., Nicola N., Begley C.G. (1997). Recombinant soluble interleukin-11 (IL-11) receptor α-chain can act as an IL-11 antagonist. Blood.

[bib10] Dahmen H., Horsten U., Kuster A., Jacques Y., Minvielle S., Kerr I.M., Ciliberto G., Paonessa G., Heinrich P.C., Muller-Newen G. (1998). Activation of the signal transducer gp130 by interleukin-11 and interleukin-6 is mediated by similar molecular interactions. Biochem. J..

[bib11] DeLano, W.L. (2002). The PyMOL Molecular Graphics System (http://www.pymol.org).

[bib12] Esnouf R.M. (1997). An extensively modified version of MolScript that includes greatly enhanced coloring capabilities. J. Mol. Graph. Model..

[bib13] Harauz G., van Heel M. (1986). Exact filters for general geometry three dimensional reconstruction. Optik.

[bib14] He W., Gong K., Smith D.K., Ip N.Y. (2005). The N-terminal cytokine binding domain of LIFR is required for CNTF binding and signalling. FEBS Lett..

[bib15] Hibi M., Murakami M., Saito M., Hirano T., Taga T., Kishimoto T. (1990). Molecular cloning and expression of an IL-6 signal transducer gp130. Cell.

[bib16] Kishimoto T., Akira S., Narazaki M., Taga T. (1995). Interleukin-6 family of cytokines and gp130. Blood.

[bib17] Kleywegt G.J., Jones T.A. (1997). Template convolution to enhance or detect structural features in macromolecular electron-density maps. Acta Crystallogr. D Biol. Crystallogr..

[bib18] Kurth I., Horsten U., Pflanz S., Timmermann A., Kuster A., Dahmen H., Tacken I., Heinrich P.C., Muller-Newen G. (2000). Importance of the membrane-proximal extracellular domains for activation of the signal transducer glycoprotein 130. J. Immunol..

[bib19] Livnah O., Stura E.A., Johnson D.L., Middleton S.A., Mulcahy L.S., Wrighton N.C., Dower W.J., Jolliffe L.K., Wilson I.A. (1996). Functional mimicry of a protein hormone by a peptide agonist: the EPO receptor complex at 2.8 Å. Science.

[bib20] Livnah O., Johnson D.L., Stura E.A., Farrell F.X., Barbone F.P., You Y., Liu K.D., Goldsmith M.A., He W., Krause C.D. (1998). An antagonist peptide-EPO receptor complex suggests that receptor dimerization is not sufficient for activation. Nat. Struct. Biol..

[bib21] Matadeen R., Patwardhan A., Gowen B., Orlova E.V., Pape T., Cuff M., Mueller F., Brimacombe R., van Heel M. (1999). The *Escherichia coli* large ribosomal subunit at 7.5 Å resolution. Struct. Fold. Des..

[bib22] Merritt E.A., Bacon D.J. (1997). Raster3D photorealistic molecular graphics. Methods Enzymol..

[bib23] Moritz R.L., Hall N.E., Connolly L.M., Simpson R.J. (2001). Determination of the disulfide structure and N-glycosylation sites of the extracellular domain of the human signal transducer gp130. J. Biol. Chem..

[bib24] Navaza J., Lepault J., Rey F.A., Alvarez-Rua C., Borge J. (2002). On the fitting of model electron densities into EM reconstructions: a reciprocal-space formulation. Acta Crystallogr. D Biol. Crystallogr..

[bib25] Orlova E.V., Dube P., Harris J.R., Beckman E., Zemlin F., Markl J., van Heel M. (1997). Structure of keyhole limpet hemocyanin type 1 (KLH1) at 15 Å resolution by electron cryomicroscopy and angular reconstitution. J. Mol. Biol..

[bib26] Paonessa G., Graziani R., De Serio A., Savino R., Ciapponi L., Lahm A., Salvati A.L., Toniatti C., Ciliberto G. (1995). Two distinct and independent sites on IL-6 trigger gp 130 dimer formation and signalling. EMBO J..

[bib27] Rosenthal P.B., Henderson R. (2003). Optimal determination of particle orientation, absolute hand, and contrast loss in single-particle electron cryomicroscopy. J. Mol. Biol..

[bib28] Sayle R.A., Milner-Whilte E.J. (1995). RASMOL: biomolecular graphics for all. Trends Biochem. Sci..

[bib29] Sharma A., Askari J.A., Humphries M.J., Jones E.Y., Stuart D.I. (1999). Crystal structure of a heparin- and integrin-binding segment of human fibronectin. EMBO J..

[bib30] Skiniotis G., Boulanger M.J., Garcia K.C., Walz T. (2005). Signalling conformations of the tall cytokine receptor gp130 when in complex with IL-6 and IL-6 receptor. Nat. Struct. Mol. Biol..

[bib31] Socolovsky M., Fallon A.E., Wang S., Brugnara C., Lodish H.F. (1999). Fetal anemia and apoptosis of red cell progenitors in Stat5a−/−5b−/− mice: a direct role for Stat5 in Bcl-X(L) induction. Cell.

[bib32] Suhre K., Sanejouand Y.H. (2004). *ElNemo*: a normal mode web server for protein movement analysis and the generation of templates for molecular replacement. Nucleic Acids Res..

[bib33] Taga T., Kishimoto T. (1997). Gp130 and the interleukin-6 family of cytokines. Annu. Rev. Immunol..

[bib34] Tama F., Sanejouand Y.H. (2001). Conformational change of proteins arising from normal mode calculations. Protein Eng..

[bib35] Tama F., Wriggers W., Brooks C.L. (2002). Exploring global distortions of biological macromolecules and assemblies from low-resolution structural information and elastic network theory. J. Mol. Biol..

[bib36] Tama F., Valle M., Frank J., Brooks C.L. (2003). Dynamic reorganization of the functionally active ribosome explored by normal mode analysis and cryo-electron microscopy. Proc. Natl. Acad. Sci. USA.

[bib37] Timmermann A., Kuster A., Kurth I., Heinrich P.C., Muller-Newen G. (2002). A functional role of the membrane-proximal extracellular domains of the signal transducer gp130 in heterodimerization with the leukemia inhibitory factor receptor. Eur. J. Biochem..

[bib38] van Heel M. (1987). Angular reconstitution: a posteriori assignment of projection directions for 3D reconstruction. Ultramicroscopy.

[bib39] van Heel M. (1989). Classification of very large electron microscopical image data set. Optik.

[bib40] van Heel M., Harauz G., Orlova E.V., Schmidt R., Schatz M. (1996). A new generation of the IMAGIC image processing system. J. Struct. Biol..

[bib41] Varghese J.N., Moritz R.L., Lou M.Z., Van Donkelaar A., Ji H., Ivancic N., Branson K.M., Hall N.E., Simpson R.J. (2002). Structure of the extracellular domains of the human interleukin-6 receptor α-chain. Proc. Natl. Acad. Sci. USA.

[bib42] Ward L.D., Howlett G.J., Discolo G., Yasukawa K., Hammacher A., Moritz R.L., Simpson R.J. (1994). High affinity interleukin-6 receptor is a hexameric complex consisting of two molecules each of interleukin-6, interleukin-6 receptor, and gp-130. J. Biol. Chem..

[bib43] Wilson I.A., Jolliffe L.K. (1999). The structure, organization, activation and plasticity of the erythropoietin receptor. Curr. Opin. Struct. Biol..

[bib44] Wriggers W., Milligan R.A., McCammon J.A. (1999). Situs: a package for docking crystal structures into low-resolution maps from electron microscopy. J. Struct. Biol..

